# SPIDR: small-molecule peptide-influenced drug repurposing

**DOI:** 10.1186/s12859-018-2153-y

**Published:** 2018-04-16

**Authors:** Matthew D. King, Thomas Long, Daniel L. Pfalmer, Timothy L. Andersen, Owen M. McDougal

**Affiliations:** 1 Department of Chemistry and Biochemistry, Boise State University, Boise, USA; 2 Department of Computer Science, Boise State University, Boise, USA; 3 Biomolecular Sciences Ph.D. Program, Boise State University, Boise, USA

**Keywords:** Drug repurposing, Repositioning, DockoMatic, GAMPMS, SimSearcher

## Abstract

**Background:**

Conventional de novo drug design is costly and time consuming, making it accessible to only the best resourced research organizations. An emergent approach to new drug development is drug repurposing, in which compounds that have already gone through some level of clinical testing are examined for efficacy against diseases divergent than their original application. Repurposing of existing drugs circumvents the time and considerable cost of early stages of drug development, and can be accelerated by using software to screen existing chemical databases to identify suitable drug candidates.

**Results:**

Small-molecule Peptide-Influenced Drug Repurposing (SPIDR) was developed to identify small molecule drugs that target a specific receptor by exploring the conformational binding space of peptide ligands. SPIDR was tested using the potent and selective 16-amino acid peptide *α*-conotoxin MII ligand and the *α*_3_*β*_2_-nicotinic acetylcholine receptor (nAChR) isoform. SPIDR incorporates a genetic algorithm-based, heuristic search procedure, which was used to explore the ligand binding domain of the *α*_3_*β*_2_-nAChR isoform using a library consisting of 640,000 *α*-conotoxin MII peptide analogs. The peptides that exhibited the highest affinity for *α*_3_*β*_2_-nAChR were used as models for a small-molecule structure similarity search of the PubChem Compound database. SPIDR incorporates the SimSearcher utility, which generates shape distribution signatures of molecules and employs multi-level K-means clustering to insure fast database queries. SPIDR identified non-peptide drugs with estimated binding affinities nearly double that of the native *α*-conotoxin MII peptide.

**Conclusions:**

SPIDR has been generalized and integrated into DockoMatic v 2.1. This software contains an intuitive graphical interface for peptide mutant screening workflow and facilitates mapping, clustering, and searching of local molecular databases, making DockoMatic a valuable tool for researchers in drug design and repurposing.

**Electronic supplementary material:**

The online version of this article (10.1186/s12859-018-2153-y) contains supplementary material, which is available to authorized users.

## Background

Conventional de novo drug development involves identifying a lead drug candidate, optimizing its structural and pharmacological properties, and then validating it through expensive and time intensive pre-clinical and clinical trials. Historically, only 1 in 10 drug candidates that enter clinical trials yields a marketable drug that is both highly effective and induces few if any undesirable side effects [1, 2]. A successful drug from concept to market costs on the order of ~$2.8 billion (USD) with an average development time of 14 years [1, 2]. As a result, the number of new drugs approved each year remains low, and the exorbitant cost of successes and failures are passed on to the consumer.

The problems with conventional de novo drug development have led the National Institutes of Health (NIH), university researchers, and pharmaceutical companies to explore ‘drug repurposing’ (aka ‘drug repositioning’) as an alternative path to drug development [3–5]. Drug repurposing jumpstarts the drug development process by using compounds that have already gone through some level of clinical testing, rather than attempting to create new unproven drugs. Drug repurposing has led to many noteworthy successes including Viagra (sildenafil), Requip (ropinirole), and Chantix (varenicline) among others. The drug-repurposing paradigm accounted for nearly 30% of United States Food and Drug Administration (FDA) approved drugs between 1999 and 2008 [6]. This achievement directly correlated to emergence of large, publicly-available chemical databases. One prominent example is the NIH PubChem Compound database which contains structural and bioactivity information for over 51 million small molecules, in addition to web-based tools for performing substructure, shape, and database searches of other publically available databases [7].

The prediction of the specific interaction of a small molecule and biological receptor is a central problem in biochemistry and pharmacology. Many software programs (e.g., WinDock [8], BDT [9], Glide [10], and DockoMatic [11, 12]) have been developed for high-throughput virtual screening (HTVS) of compound libraries that take advantage of rapid mathematical methods for predicting the interaction strength between two bound molecules of a given orientation. The challenge that remains is prediction of the binding orientation for two molecules, a process that requires each molecule of the binding pair to come together in a variety of conformations to identify the optimal partnership [7].

DockoMatic [11, 12] is an open source software meta-tool consisting of a graphical user interface that employs AutoDockTools and AutoDock 4.2 to facilitate set-up, calculation, and result analysis for large numbers of docking jobs [13, 14]. In addition to single ligand/receptor docking, DockoMatic can be used for secondary ligand docking, peptide ligand structure creation with Obconformer [15], and in silico site-directed mutagenesis of peptide or protein structures with TreePack [16, 17]. DockoMatic was originally developed to facilitate the creation of a library of mutated peptides for docking to a multi-subunit protein receptor without manually generating the mutated peptide structures.

In the natural world, some of the most potent inhibitors/initiators of biological functions take the form of small peptides, including many variations found in the venom of some spiders, wasps, snakes, and marine snails [18]. These effective and highly specific biomolecules have received significant attention by the scientific community due to their demonstrated translation to therapeutic treatments for a variety of afflictions including pain (Prialt), hypertension (angiotensin-converting enzyme ‘ACE’ inhibitors), Type 2 diabetes (Exenatide), and malignant glioma (chlorotoxin TM-601) [19, 20]. However, peptide-based pharmaceuticals have been marginally adopted due to their rapid degradation by gastrointestinal enzymes, making administration of the drugs challenging. Identification of small molecules with similar shape and pharmacophore features to those of bioactive peptides will lead to development of orally-available biomimetic drugs with analogous pharmacological actions.

nAChRs are pentameric ligand-gated ion channels critically important in neuronal survival and cognitive function, and regulation of neurodegenerative diseases, including Alzheimer’s and Parkinson’s [21–25]. *α*-Conotoxins (*α*-CTxs) are small (10–30 residue) peptides derived from the venom of predatory marine cone snails of the genus *Conus* that discriminate between nAChR isoforms [26–29]. Their bioactive specificity and potency has led to *α*-CTxs being used as molecular probes to determining the structure/function relationships of nAChRs, and has the potential to lead to significant advancements in the pharmacology of neurodegenerative disorders [30].

**Fig. 1:**
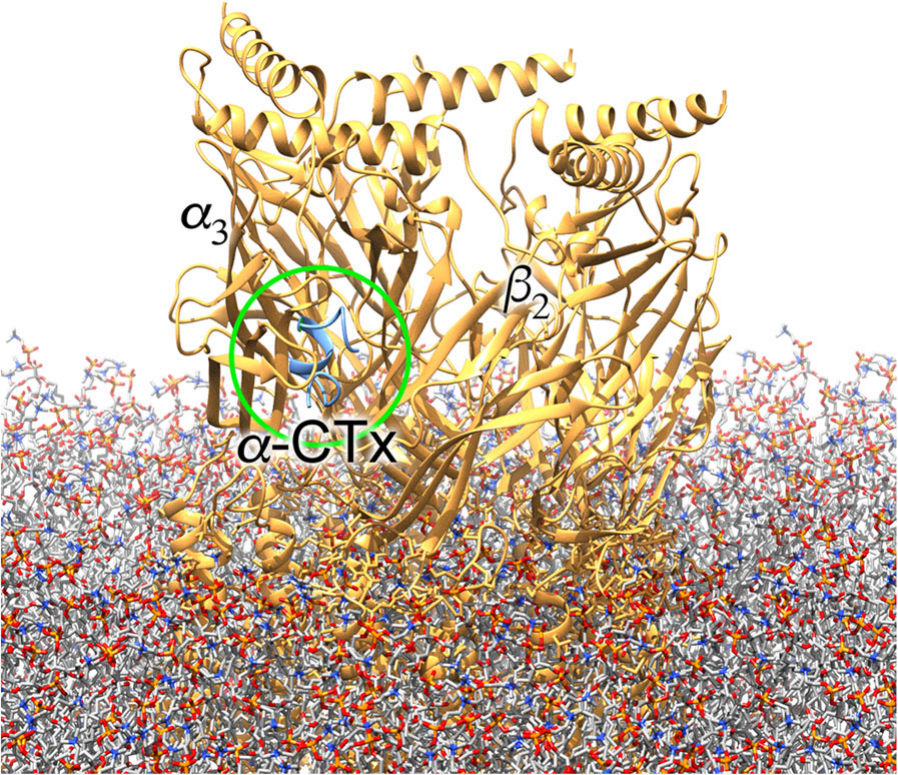
*α*-CTx MII bound to the transmembrane ligand-gated ion channel *α*_3_*β*_2_-nAChR. Note that native receptor is a pentamer, whereas computational modeling utilizes a dimer consisting of known binding site for *α*-CTxs between *α*_3_- and *β*_2_-subunits

*α*-CTx MII is a 16 amino acid peptide with an *IC*_50_ of 0.5 nM for the *α*_3_*β*_2_-nAChR isoform [26]. Binding of *α*-CTx MII with *α*_3_*β*_2_-nAChR occurs between the *α*_3_- and *β*_2_-subunits, with the peptide docking in the large pocket under the C-loop of the *α*_3_-subunit (Fig. 1). Site directed mutagenesis studies on nAChRs, investigations into the alteration of the primary sequence of *α*-CTx MII, and molecular modeling approaches have all been conducted to help understand the selectivity and potency of *α*-CTx MII and its variants [31–33]. In this study, the small-molecule peptide-influenced drug repurposing (SPIDR) workflow was developed to survey *α*-CTx MII peptide analogs that most favorably bind *α*_3_*β*_2_-nAChRs, and extrapolate complementary atomistic contacts to small molecule drugs exhibiting the desired qualities identified by screening drug repurposing databases. SPIDR executes the following three steps: 1) perform a structure-based high-throughput virtual screening of an *α*-CTx MII mutant library to find peptides with high binding affinity for the *α*_3_*β*_2_-nAChR; 2) use these peptide structures to perform a ligand-based survey of the PubChem Compound database to identify FDA approved drugs with 3-D conformations similar to the high affinity peptides; and 3) perform molecular docking calculations between the resulting small molecule drugs and the *α*_3_*β*_2_-nAChR.

## Results and discussion

The first step in the SPIDR workflow uses genetic algorithm managed peptide mutant screening (GAMPMS) [34] to perform a comprehensive structure-based screen of a peptide mutant library. GAMPMS implementation required a total of 9344 molecular docking jobs to explore 640,000 variants of *α*-CTx MII. Sequences of the peptide mutants found to have the highest binding affinities are shown in Table 1. The estimated binding free energy of *α*-CTx MII was − 12.38 kcal/mol compared to the ∆*G*_bind_ of the top ten mutants ranging from − 20.66 to − 21.07 kcal/mol, indicating that the analog screening process identified peptide ligands with more favorable receptor binding energies than the native peptide. High sequence similarity was observed for the best *α*-CTx MII mutants. Notably, each mutant contained the residues Tyr5 and Trp10 in place of the *α*-CTx MII residues Asn5 and Leu10, respectively, as well as a His-12-Ser mutation in 80% of the mutants. The His9 residue of *α*-CTx MII was conserved in half of the top mutant sequences. A more robust treatment of GAMPMS results for predicted conotoxin mutant binding to the α3β2-nAChR isoform can be found in ref [35].

**Table 1:** The 10 highest affinity peptides found with GAMPMS compared with the native *α*-CTx MII peptide for binding with *α*_3_*β*_2_-nAChR

Peptide^*a*^	∆*G*_bind_^*b*^
GCCS **Y** PVC **YWTN** SNLC	−21.07
GCCS **Y** PVCH **WQS** SN **F** C	− 20.91
GCCS **Y** PVC **YWQS** SN **V** C	−20.91
GCCS **Y** PVCH **WSS** SN **F** C	−20.88
GCCS **Y** PVCH **WSS** SN **W** C	−20.79
GCCS **Y** PVC **SWKS** SN **F** C	−20.74
GCCS **Y** PVCH **WYS** SN **V** C	−20.73
GCCS **Y** PVC **KWSN** SN **G** C	−20.71
GCCS **Y** PVC **NWSS** SN **W** C	−20.68
GCCS **Y** PVCH **WKS** SN **G** C	−20.66
GCCSNPVCHLEHSNLC (MII)	−12.38

^***a***^Mutations in bold type; ^***b***^kcal/mol, estimated in AutoDock

The high sequence similarity and comparable estimated binding affinities of the top mutants indicate that the residues in bold print in Table 1 are critical in the formation of significantly more favorable interactions in the ligand-receptor complex compared to native *α*-CTx MII. These favorable attributes are also advantageous when using these sequences as templates for searching small molecules that may form the same types of ligand-receptor interactions.

The new SimSearcher utility, developed using the *α*_3_*β*_2_-nAChR system, allows for rapid similarity searches with any target molecule of any size and conformational flexibility over local molecular databases. The management of SimSearcher employs an intuitive graphical interface in DockoMatic 2.1 to proceed through the Map, Cluster, and Search steps. Development of additional signature types and corresponding similarity metrics could increase SimSearcher’s utility. A pharmacophore signature and corresponding similarity metric have been created and are included in DockoMatic 2.1, but pharmacophore clustering is not yet supported. The additions to DockoMatic 2.1 resulting from this work have greatly improved the software’s capabilities and efficacy as a powerful tool for exploring receptor conformational binding space with peptide mutant analogs and identification of small molecules as potential lead compounds for drug repurposing. We sought to evaluate the usefulness of SimSearcher and considered databases including DrugBank, BindingDB, Chem Spider, ChEMBL, and PubChem [36–40]. Of these resources PubChem offered the greatest variation and number of molecules.

To evaluate the efficacy of clustering the signature database before performing a comprehensive similarity search using SimSearcher, the Cluster and Search steps were initially tested with a single target molecule (CID 1, where CID is the PubChem compound identifier) for the 10 most similar molecules [40]. This was done by two comparative searches, one using the entire collection of generated PubChem compound signatures, and the other using multilevel K-means clustering of the signatures. For clustering, a χ^2^ test was used to assess the distance between signatures. As a result of the clustering, the signatures were divided into 50 clusters, each containing 20 subclusters, and each subcluster containing 5 sub-subclusters. The search of the non-clustered signature database took approximately 24 min to complete and performed on the order of 51 million similarity calculations. By comparison, the multilevel K-means clustering search required only a few seconds, and performed far fewer similarity calculations (~ 15,000). In both searches, the same resulting 10 molecules were identified.

**Fig. 2:**
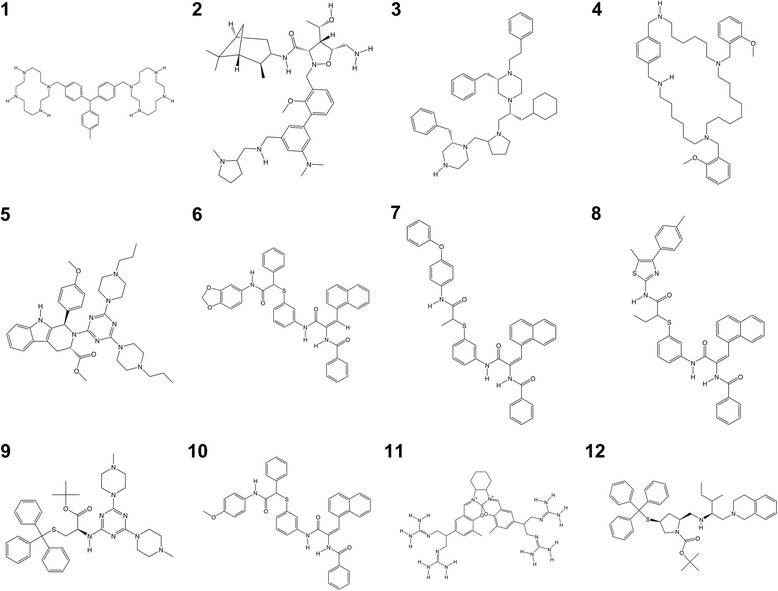
The 12 small molecules from the PubChem Compound database with the predicted highest binding affinity for the *α*_3_*β*_2_-nAChR isoform. PubChem CIDs for the above compounds are provided in Table 2

The 20,000-molecule clustered signature database was queried with the top 200 peptides from GAMPMS. Duplicate molecules and those containing silicon, which is not parameterized in the AutoDock scoring function, were removed from the collection, leaving only 1320 molecules. Each of these potential drug molecules was docked against the *α*_3_*β*_2_-nAChR model using AutoDock with 40 pose evaluations. The 1320 molecules were then re-clustered and the molecule with the highest binding affinity with the *α*_3_*β*_2_-nAChR was selected from each cluster. In this manner, the top 128 molecules were identified. The 12 molecules with the highest predicted binding affinity to *α*_3_*β*_2_-nAChR from the set of 128 are shown in Fig. 2. The CIDs, molecular formula, molecular mass, and AutoDock scores for the 12 molecules are provided in Table 2. Each of the small molecules had a more favorable predicted binding free energy than that of *α*-CTx MII (Δ*G*_bind_ = − 12.38 kcal/mol). The top small molecule candidate had an estimated Δ*G*_bind_ = − 21.88 kcal/mol, which was slightly more favorable than the Δ*G*_bind_ = − 21.07 kcal/mol predicted for the best peptide mutant.

**Table 2:** The 12 small molecules from the PubChem Compound database with the highest predicted binding affinity for *α*_3_*β*_2_–nAChR identified by SPIDR [41–52]

Rank	CID^*a*^	Molecular Formula	Molar Mass^*b*^	∆*G*_bind_^*c*^
1	25,131,416	C_42_H_66_N_8_	683.03	−21.88
2	58,420,086	C_40_H_62_N_6_O_4_	690.96	−17.87
3	46,883,273	C_44_H_63_N_5_	662.00	−17.32
4	11,017,883	C_44_H_68_N_4_O_2_	685.04	−17.19
5	46,702,076	C_37_H_49_N_9_O_3_	667.84	−16.20
6	19,311,642	C_41_H_31_N_3_O_5_S	677.77	−16.02
7	19,311,407	C_41_H_33_N_3_O_4_S	663.78	−15.92
8	19,303,632	C_41_H_36_N_4_O_3_S_2_	696.88	−15.62
9	69,091,626	C_39_H_50_N_8_O_2_S	694.93	−15.55
10	19,311,613	C_41_H_33_N_3_O_4_S	663.78	−15.55
11	58,320,126	C_33_H_48_N_14_O_2_^2+^	672.83	−15.50
12	67,754,078	C_44_H_55_N_3_O_2_S	689.99	−15.40

^***a***^PubChem compound identifier; ^***b***^g/mol; ^***c***^kcal/mol

Similarities were observed in the chemical structures of the top 12 small molecules. Each consists of multiple ring structures and an associated large surface area that is compatible with the relatively high number of hydrophobic residues in the *α*_3_*β*_2_-nAChR binding pocket. All of the top molecules are amine-rich; all but one (compound **11**) have a secondary amine capable of acting as either a hydrogen bond donor or acceptor. However, most of the moieties available for hydrogen bonding in these molecules would act as acceptors, with high numbers of tertiary amines, carbonyl and ether groups. Many of the compounds have similar structural components, most notably compounds **6**, **7**, **8**, and **10**, which have the same base structure with variations in the ringed addition linked through the thiophenol groups. Compounds **6** and **10** differ only in the elimination of a single oxygen atom (and addition of two hydrogen atoms) in the terminal five-member ring of compound **6**. Another pair of like compounds are **9** and **12**, which share the same base structure. The sizes of the top compounds are comparable with molecular masses in the range of 662–697 Da, which is much smaller than the molecular mass of native *α*-CTx MII (~ 1711 Da), although relatively large when considering small drug-like molecules. ‘Larger’ small molecules with greater surface area, such as ring-containing compounds, are more likely to correlate to the peptide signatures when associating with the sizable binding region of nAChR.

**Fig. 3:**
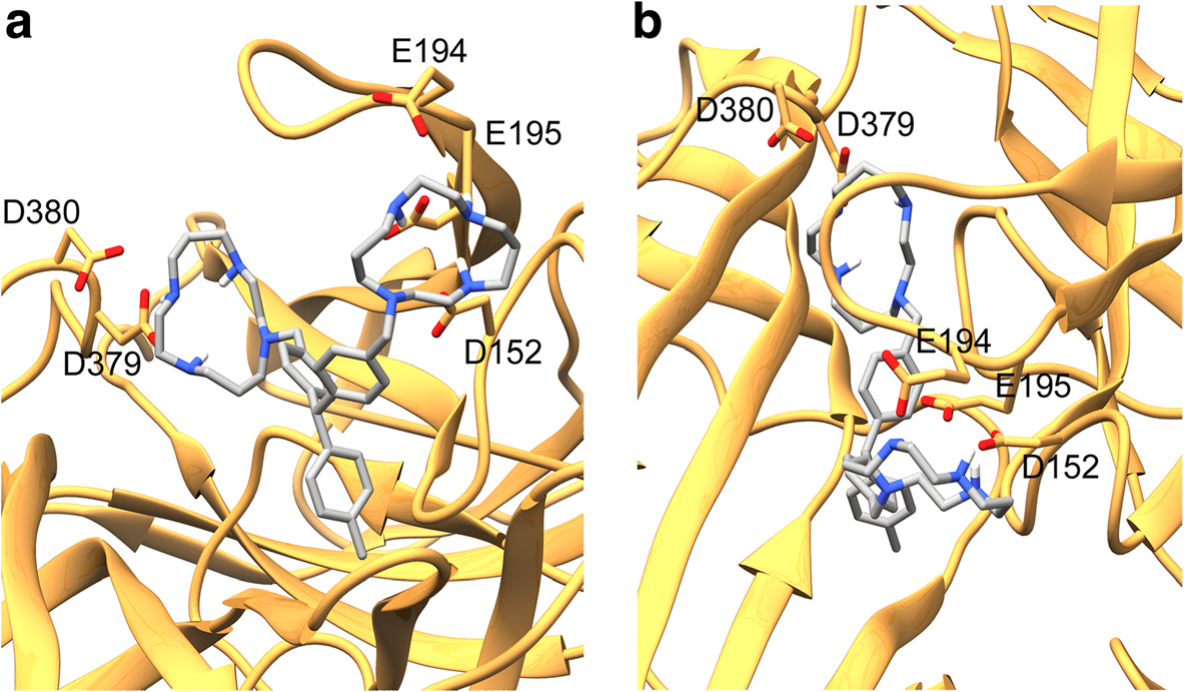
Binding orientation of the highest binding affinity small molecule **1** (CID: 25131416) with *α*_3_*β*_2_-nAChR predicted by molecular docking, where panel A provides one view of the ligand-receptor complex with the C-loop on top, and B represents the perspective looking through the C-loop

The high affinity of molecule **1** with *α*_3_*β*_2_-nAChR is largely due to the strong electrostatic interactions between amine moieties and receptor Asp and Glu residues containing charged carboxyl groups (Fig. 3). The length of the molecule spans the binding pocket with each of the amine-containing ring structures interacting with a distinct concentration of negatively charged residues on separate subunits. The Glu194 and Glu195 residues belonging to the *α*_3_-subunit are part of the C-loop, the dynamics of which are critical in the functionality of nAChRs [53–55]. Interrupting the opening/closing of the C-loop by **1** could render this molecule a potent antagonist (or agonist) to normal function of nAChRs. Since the precise mechanism of activation of nAChRs remains unclear, the effects of small molecule binding are unknown; although it is likely that **1** would have strong antagonist action on nAChR since it was modeled after potent α-CTx antagonists. In addition to the strong coulombic interactions observed in the binding of **1** to *α*_3_*β*_2_-nAChR subunits, there are also significant apparent hydrophobic contributions between the aromatic ring portion of the molecule and hydrophobic residues in the deep binding pocket of nAChR. The combination of favorable interactions is reflected in the predicted high binding affinity for this molecule.

The design of this study was to demonstrate proof-of-principle of the developed SPIDR workflow to identify potential drug candidates for repurposing based on mapping of the conformational binding space of small peptide ligands with a target receptor. As such, detailed pharmacokinetic profiles of the top small-molecule candidates were not created in this study. This is, however, an important aspect of drug development and repurposing. Fortunately, many useful tools are available for quickly identifying important pharmacokinetic properties, including potential toxicity, absorption, distribution, metabolism, and excretion, which aid in determining the potential efficacy of a drug candidate upon administration to a patient. Online servers, such as admetSAR [56], SwissADME [57], and OCHEM [58, 59], allow users to submit chemical structures and retrieve pharmacokinetic and physical properties relating to drug-like characteristics and potential biological activities. This provides researchers knowledge of deficiencies in drug design and performance, and expedites the drug development and repurposing process by either eliminating potentially ineffective candidates or identifying modifications to the compound that can improve pharmacological characteristics [60–62]. Future development of the DockoMatic software package will include the capability for pharmacokinetic analysis of drug candidates.

## Methods

The DockoMatic software package and the integration of GAMPMS is described in detail elsewhere [10, 11, 33]. The receptor structure used in GAMPMS was a homology model of the *α*_3_*β*_2_-nAChR isoform constructed from the amino acid sequences of *α*_3_- (UniProtKB: P04757.1) and *β*_2_- (UniProtKB: P12390.2) subunits of rat neuronal nAChR and using the *Torpedo marmorata* nAChR (PDB ID: 2BG9) as a structural template [63, 64]. The homology models were created using the DockoMatic 2.1 and MODELLER packages [65]. The *α*_3_*β*_2_-nAChR subunit dimer consisting of only the extracellular domains, although nAChRs exist naturally as a pentameric transmembrane protein complexes [27].

**Fig. 4:**
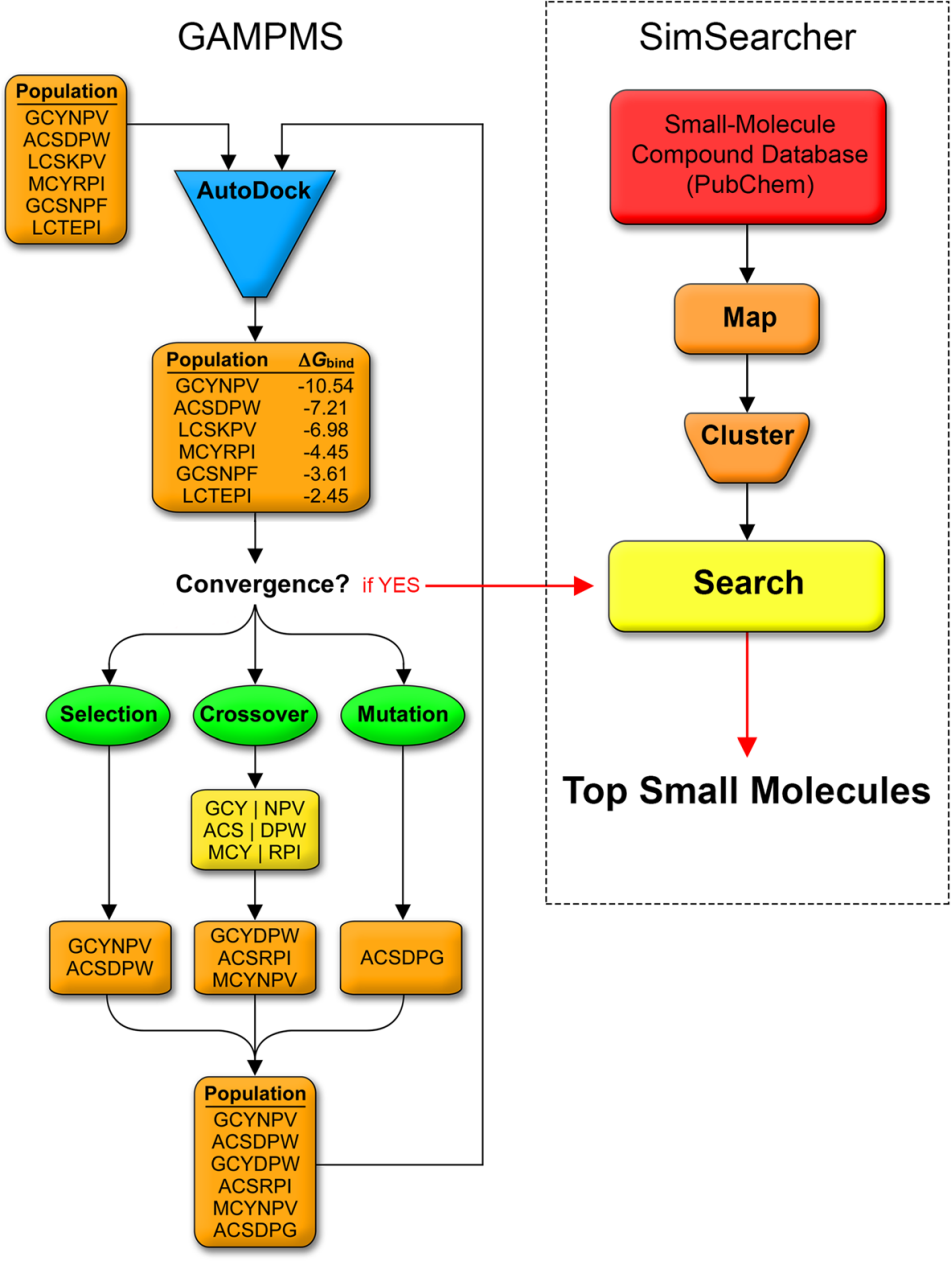
Schematic representation of the SPIDR workflow using the GAMPMS and SimSearcher utilities found in DockoMatic 2.1

PubChem’s file transfer protocol (FTP) tool was used to download the most diverse conformer for each molecule in the PubChem Compound database. The directory contained 2864 spatial data files (SDFs), with each covering a range of 25,000 CIDs. The total number of structures screened using SPIDR was approximately 51 million. The workflow of SPIDR, which includes the GAMPMS and SimSearcher utilities of DockoMatic 2.1, is shown in Fig. 4 and described in detail below.

### GAMPMS

The peptide mutant library was defined as the native *α* − *CTx* MII peptide sequence and a set of mutation constraints. *α-*CTx MII has the primary sequence GCCSNPVCHLEHSNLC, with two disulfide bonds between Cys2-Cys8 and Cys3-Cys16, and features an *α*-helix spanning from Pro6 to His12. Mutation constraints specify which residues are subject to mutations and which amino acids can be substituted for each mutable residue. The approach to generating the 640,000 *α*-CTx MII mutant ligand library is defined in Table 3. Six residues: Asn5, His9, Leu10, Glu11, His12, and Leu15, were considered mutable. The residues important to initiating the *α*-helix (i.e., Pro6) or maintaining structural stability (i.e., Cys2, Cys3, Cys8, and Cys16) were left unchanged. Both polar/charged and nonpolar residues were constrained to mutations to residues of like character. The possible combinations of amino acid substitutions in the mutation space results in a total of 640,000 different peptide sequences. A detailed description of the GAMPMS methodology can be found in ref. [34].

**Table 3:** The *α* − *CTx* MII mutant ligand library defined as a base peptide and a set of mutation constraints

Mutable Residue	Substitutable Amino Acids
N 5	S T Y N Q D E K R H
H 9	S T Y N Q D E K R H
L 10	G A V L I M W F
E 11	S T Y N Q D E K R H
H 12	S T Y N Q D E K R H
L 15	G A V L I M W F

Each individual was represented as a character array using single-letter amino acid identifiers. The fitness of an individual was evaluated by first constructing the peptide analog through a set of residue mutations to the base peptide, followed by molecular docking against the target receptor. The estimated binding free energy for the highest affinity pose produced by the AutoDock scoring function was considered the fitness value for the individual. The user-defined **elitism** operator was used to select the top fraction of the most fit mutants of a population to be passed on to the successive population. A two-parent, two-offspring, *N*-point **crossover** was used as a fitness-proportionate selection scheme. Two top results from the current population were selected with a probability directly proportional to their fitness ranking. The two parents were split into *N* + 1 regions that were alternated to make two different offspring sharing features of both parents. The **mutation** operator provided an amino acid an equal chance of being substituted for any other amino acids within the defined set shown in Table 3. The resulting next-generation populations were used as subsequent input sequences for docking until the convergence criteria were achieved. New populations were generated by GAMPMS until reaching the specified convergence criteria. The genetic algorithm was terminated when there was no change in the *top X* highest affinity peptides over the last *λ* iterations, both parameters were specified in the DockoMatic 2.1 workflow.

The screening was performed on the Fission high-performance computing cluster located at Idaho National Laboratory, Idaho Falls, ID. Forty pose evaluations were used in the AutoDock docking simulation for ligand-receptor binding. A total of 9344 molecular docking jobs were performed as 73 groups of 128 jobs (over 128 cores). GAMPMS was configured to carryover the top 40% of each population, use a two-parent, two-offspring, three-point crossover, and have a 2% residue mutation probability. The GA terminated after 5 rounds without an improvement in the binding affinity of the 50 top peptides.

### Drug similarity search

After identifying a set of *α*-CTx MII mutants with a high binding affinity to *α*_3_*β*_2_-nAChR by GAMPMS, small-molecular-weight drugs from the PubChem Compound database were searched for those closely resembling the 3-D shapes of the peptide ligands. Although the PubChem online search tools include similar functionalities, limitations of these tools prevent screening against the peptide library.

Generating the fingerprint for every molecule is a computationally demanding endeavor, but the fingerprints could be pre-computed in a highly parallel manner. However, determining reference shapes and generating fingerprints for peptides requires an excessive amount of time and would have limited the input sequences. Instead, a shape distribution technique was used to assess 3-D shape similarity between molecules [66, 67]. With shape distribution, a shape sampling function is used to construct a distribution of measurements. The distribution serves as the molecule signature, and a distribution difference measure, such as the *χ*^2^ test, is used to quickly compare the signatures. To reduce the time to perform distribution tests for 51 million compounds, multilevel K-means clustering was implemented. This allowed a recursive search operation to compare the target molecule with a clustered subset, thus reducing the number of comparisons required for each search.

The following model was developed for similarity searches with any target molecule over local molecular databases. For clarity, using a molecule *M* as the basis of a similarity search (i.e. searching with a target molecule *M*) over a database *D* is equivalent to searching *D* for items which are similar to *M*. The model consisted of three steps:

1. ***Map*** **–** Map all molecules to signatures
2. ***Cluster*** **–** Cluster the signatures for expedited searching
3. ***Search*** **–** Map the target molecule to a signature, search the (clustered) database for similar signatures.

Signature mapping must first be performed for tractable searching. The Cluster step is optional but can be used to reduce search time by several orders of magnitude. The Map and Cluster steps are computationally expensive but only need to be performed once per database and can be pre-computed. Search is the end product of the process, allowing users to quickly perform molecular similarity searches over the databases.

Generating signatures is a highly parallel problem that is made simpler by the fact that molecular databases are typically downloaded as a collection of data files. To quickly generate signatures, it is necessary to first partition the database files to create a partition for each available processing core. Then, using a function to generate a signature for a molecule, an instance of the mapping algorithm can be run on each processor in order to generate signatures for the associated partition. The signature needs to be both descriptive and easily comparable so that a similarity metric can be discriminative and efficient, respectively. Signatures can be precomputed (offline), making the computational complexity of their generation less important than that of the similarity metric.

The shape distribution was used to gauge the 3-D shape similarity of two molecules. In this approach, a shape sampling function was applied to a 3-D shape in order to attain a set of measurements. The distribution of these measurements was used as the shape signature. Any distribution difference test (e.g. *χ*^2^) could be applied to the two signatures to quickly judge the similarity of the associated molecules. This approach has been successfully applied to compare 3-D protein structures [68]. The implemented shape sampling function measures the Euclidean distance between unique pairs of atoms within a molecule. The computational need for sampling was configured by defining the number of samples. Since most of the molecules within PubChem Compound are small (less than 50 atoms), it was feasible to generate a distribution using all $\frac{\mathrm{N}\left(\mathrm{N}+1\right)}{2}$ unique measurements, with *N* representing the number of atoms in the molecule. The distribution is represented as a histogram containing a constant number of bins and a maximum measurement threshold. **Algorithms 1** and **2** demonstrate the process used to create a molecule shape signature. **Algorithm 2** was used to generate shape signatures for a group of data files. Four similarity metrics were implemented for signature comparison: Chi Square, L1-norm, L2-norm, and the Root of Products test.

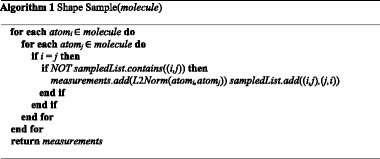

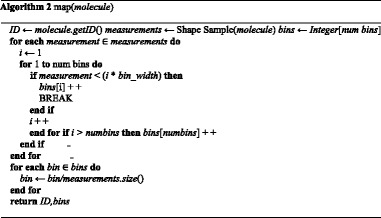





Clustering is an optional step, although it is highly recommended for shape-based similarity searches. Without clustering, searching a database with molecule *q* requires comparing the signature of *q* and every signature in the database. For the PubChem database, this would mean performing 51 million calculations. Clustering the signatures reduces the number of similarity calculations by orders of magnitude.

For example, when dealing with a database containing |*DB*| signatures, if the database is clustered with the K-means algorithm, where *K* = *k*_1_ × *k*_2_ × *...* × *k*_*n*_, then an effective search could be performed with

2 $$ \approx \mathrm{K}+\frac{\left|\mathrm{DB}\right|}{\mathrm{K}} $$

similarity calculations by comparing the target molecule to each of the *K* cluster centers and then to each of the $\frac{\left|\mathrm{DB}\right|}{\mathrm{K}}$ signatures within the cluster whose signature was most similar to the target molecule. If |DB| ≫ K, a single K-means clustering would reduce the number of comparisons by a factor of K.

Nested (multilevel) clustering can be used to further reduce search time. In multilevel clustering, most clusters contain subclusters. **Algorithm 3** gives a pseudo code algorithm for the idea, with a user calling *NlevelCluster*(*N,DB*) to perform *N* level clustering with the K-means clustering algorithm. A “Big Data” implementation of the K-means clustering algorithm was used for generating the two outermost clusters, whereas an in-memory implementation was used for subsequent clusters (See Additional file 1).

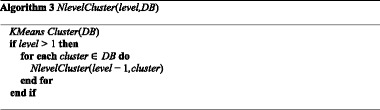



If the *DB* database is clustered with *n*-level clustering, where level *i* has *k*_*i*_ clusters (recall *K* = *k*_1_ × *k*_2_ × *...* × *k*_*n*_ from above), then the approximate number of similarity calculations required for an effective search is given by:

3 $$ \approx \sum \limits_{i=1}^n{k}_i+\frac{\left| DB\right|}{K} $$

As a result, the difference in the number of required signature calculations between the *n*-level clustering and the single clustering is given by:

4 $$ \prod \limits_{i=1}^n{k}_i-\sum \limits_{i=1}^n{k}_i $$

So if |*DB*| = 50 million and *K* = 20 × 20 × 20 = 8000, then multilevel clustering can reduce the search time by ≈ 65% compared to a single *K*-means clustering.

The idea used in the single level cluster search can be easily extended to handle nested clusters. **Algorithm 4** shows a recursive technique which can search a collection of signatures that have been subjected to N-level clustering. To search with the target molecule *q*, one would call *Search*(*q,DB*).

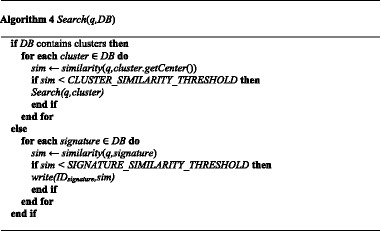



A tool to perform quick similarity searches over local molecular databases, SimSearcher, has been implemented in DockoMatic 2.1, allowing the user to perform mapping, clustering, and searching of the compound databases. In this study, the top 200 peptides from GAMPMS were used as the target molecules in the database search of the PubChem Compound library. Shape distributions, or signatures, were created for each of the 51 million small molecules in the PubChem database. The 2864 SDFs, each covering up to 25,000 CIDs, were obtained using PubChem’s FTP tool. The SDFs were divided into 16 groups of 179 files and signatures were generated for each group in parallel. For the shape distributions, Euclidean distance between all unique atom pairings within a molecule was used to sample the 3-D shape of the molecules. The distances were binned to create a histogram distribution. Each histogram contained 10 bins, and each bin had a width of 1.5 units. Distances greater than 15 units were placed in the last bin. The signature generation required approximately 3 h with highly parallel processing, with output of a signature file corresponding to each SDF. The signatures were clustered before performing the similarity search. For *N*-level K-means clustering, a χ^2^ test was used to assess the distance between signatures.

## Conclusions

Small-molecule peptide-influenced drug repurposing, SPIDR, was developed to explore the conformational ligand binding space of the *α*_3_*β*_2_-nAChR isoform and use the results to identify small molecule drugs that target the receptor. The genetic algorithm-based search procedure, GAMPMS, was used to heuristically explore the ligand binding domain of the *α*_3_*β*_2_-nAChR isoform using a 640,000 *α*-CTx MII mutant library. The GAMPMS required only 9344 docking calculations and identified peptides with estimated binding affinities 70% higher than native *α*-CTx MII. In SPIDR’s repurposing step, the PubChem Compound database was searched for molecules bearing a shape similar to the highest affinity *α*-CTx MII mutants. To perform the search with small molecules, the shape distribution-based signatures were generated for each molecule. The signatures were clustered using multilevel K-means clustering and searched with the highest affinity peptide mutants exhibiting preferred binding characteristics to the nAChR. The estimated binding affinity of the top identified small molecule (− 21.88 kcal/mol) was nearly double that of the native *α*-CTx MII peptide (− 12.38 kcal/mol).

SPIDR has been generalized and integrated with DockoMatic 2.1. DockoMatic 2.1 contains an intuitive graphical interface for a peptide mutant screening workflow, allowing a researcher to quickly create virtual peptide mutant libraries. The user has the option to screen the peptide mutant library exhaustively or with an implementation of GAMPMS. DockoMatic 2.1 also contains the SimSearcher module, which facilitates the mapping, clustering, and searching of local molecular databases. Searching a clustered database with SimSearcher requires only a few seconds per target molecule, and can accept lists of target molecules to automate larger searches. As a result, DockoMatic is a powerful tool for researchers interested in drug repurposing.

## Additional file

Additional file 1: In-Memory and Big Data Implementation of K-Means Clustering. Algorithms describing in-memory K-Means clustering of data points, and “big data” implementation of K-means clustering on a parallel computing infrastructure. (DOCX 89 kb)

## Abbreviations

CID: PubChem compound identifier
GAMPMS: Genetic algorithm managed peptide mutant screening
HTVS: High-throughput virtual screening
nAChR: Nicotinic acetylcholine receptor
SPIDR: Small-molecule peptide-influenced drug repurposing
α-CTxs: Alpha conotoxins
