# Supplementary material for: SPIDR: small-molecule peptide-influenced drug repurposing
Source: BMC Bioinformatics. 2018 Apr 16;19:138. doi: 10.1186/s12859-018-2153-y (PMC5902895; doi:10.1186/s12859-018-2153-y)
Supplement: Supplementary file 1 — In-Memory and Big Data Implementation of K-Means Clustering. Algorithms describing in-memory K-Means clustering of data points, and “big data” implementation of K-means clustering on a parallel computing infrastructure. (DOCX 89 kb) [file 12859_2018_2153_MOESM1_ESM.docx]

**In-Memory and Big Data Implementation of K-Means Clustering**

**Algorithm S1** In Memory *KMeans Cluster*(*DB,K*)

*clusters* ← choose *K* random points as cluster centers **for** *i*

∈ [0*,X*] **do**

**for each** *point* ∈ *DB* **do**

*distance* ← *MAX V ALUE*

**for each** *cluster* ∈ *clusters* **do**

*tmp* ← *getDistanceBetween*(*cluster.center,point*) **if** *tmp < distance* **then**

*closest* ← *cluster distance* ← *tmp*

**end if**

**end for**

*closest.addPoint*(*point*) **end for for each** *cluster* ∈ *clusters* **do** *distance* ← *MAX V ALUE*

**for each** *point* ∈ *cluster.getPoints*() **do**

*run total*+ = *point.coordinates*

**end for**

*cluster.setCenter*(*run total/cluster.points.size*) **end for end for**

**Algorithm S2** Big Data *KMeans Cluster*(*DB,cores,K*)

*clusters* ← choose *K* random points as cluster centers write *clusters.getCenters*() to *centers_f_ile partitions* ← *partition*(*DB, cores*) **for** *i* ∈ [0*,X*) **do**

**for each** *partition* ∈ *partitions* **do**

spawn *worker*(*partition,false*) process

**end for** wait for all workers to finish **if** i ¡ X **then**

**for each** *file* ∈ worker totals file **do**

**for** *j* ∈ [1*,K*] **do**


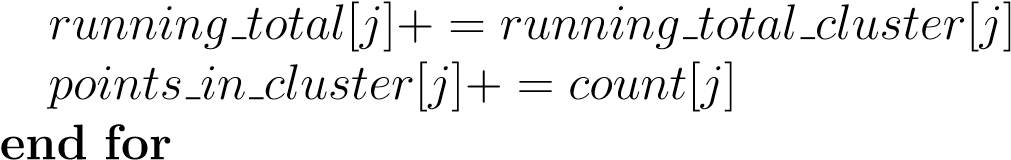


**end for for** *j* ∈ [1*,K*] **do**

*cluster*[*j*]*.setCenter*(*running total*[*j*]*/count*[*j*]) **end for**

write *clusters.getCenters*() to *centers_f_ile*

**end if**

**end for for each** *partition* ∈ *partitions* **do**

spawn *worker*(*partition,true*) process **end for**

**Algorithm S3** *worker*(*partition,final*)

*clusters* ← read from *centers_f_ile* **for**

*file* ∈ *partition* **do**

**for each** *point* ∈ *file* **do**

*distance* ← *MAX V ALUE*

**for each** *cluster* ∈ *clusters* **do**

*tmp* ← *getDistanceBetween*(*cluster.center,point*) **if** *tmp < distance* **then**

*closest* ← *cluster distance* ← *tmp*

**end if**

**end for if** *final* **then**

*closest.addPoint*(*point*)

**else**


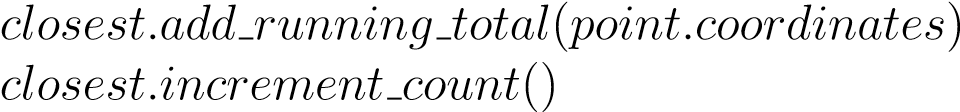


**end if**

**end for**

**if**
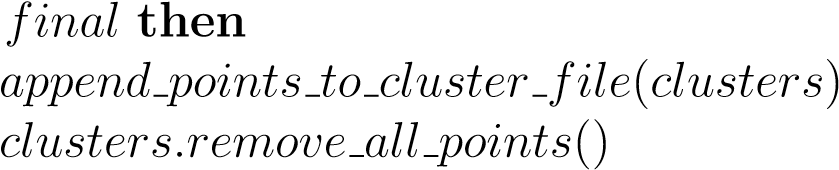


**end if**

**end for if** 6 *final* **then**

write *clusters.running totals* and *clusters.counts* to worker’s totals file **end if**
